# Multisession radiosurgery for intracranial meningioma treatment: study protocol of a single arm, monocenter, prospective trial

**DOI:** 10.1186/s13014-020-1478-7

**Published:** 2020-01-30

**Authors:** V. Pinzi, M. Marchetti, E. De Martin, V. Cuccarini, I. Tramacere, F. Ghielmetti, M. L. Fumagalli, C. Iezzoni, L. Fariselli

**Affiliations:** 1 Neurosurgery Department, Radiotherapy Unit, Fondazione IRCCS Istituto Neurologico Carlo Besta, Milan, Italy; 2 Health Department, Fondazione IRCCS Istituto Neurologico Carlo Besta, Milan, Italy; 3 Unit of Neuroradiology, Fondazione IRCCS Istituto Neurologico Carlo Besta, Via Celoria 11, Milan, Italy; 4 Department of Research and Clinical Development, Scientific Directorate, Fondazione IRCCS Istituto Neurologico Carlo Besta, Milan, Italy

**Keywords:** Radiosurgery, Multisession radiosurgery, Hypofractionated stereotactic radiotherapy, Intracranial meningioma, Large meningioma, Optic pathway meningioma, Cavernous sinus meningioma, Fractionated radiosurgery, Neurological function preservation

## Abstract

**Background:**

Single session radiosurgery represents a widely accepted treatment for intracranial meningiomas. However, this approach could involve a high risk of treatment-related complications when applied to large volume lesions. In these cases and for those not suitable for surgical resection, radiosurgery in multisession setting could represents a viable option. The literature results are reassuring in terms of correlated adverse events as well as in terms of tumor control. However, no prospective long-term results are available. In this scenario, we design a prospective monocentric phase II study, in order to verify the safety of a multisession radiosurgery schedule delivering 25 Gy in 5 daily fractions.

**Methods:**

Patients diagnosed with large and/or near to critical structures, intracranial meningiomas have been treated by means of multisession radiosurgery in both exclusive and postoperative settings. The primary study aim is safety that has been being prospectively scored based on international scales, including NCI Common Toxicity criteria, version 4.03, Barrow Neurological Institute pain intensity score, Barrow Neurological Institute facial numbness score and House-Brackmann Facial Nerve Grading System for qualitative analysis. Secondary aim is treatment efficacy in terms of local control that has been being assessed on volumetric analysis.

**Discussion:**

This is the first prospective phase II trial on multisession radiosurgery for large and/or near to critical structures intracranial meningiomas. If positive results will be found, this study could represent the starting point for a phase III trial exploring the role of multisession radiosurgery in the exclusive and postoperative radiation therapy treatment of intracranial meningiomas.

**Trial registration:**

Trial registration: clinicaltrials.gov platform (Multisession Radiosurgery in Large Meningiomas –MuRaLM- identifier NCT02974127). Registered: November 28, 2016. Retrospectively registered, https://clinicaltrials.gov/ct2/show/NCT02974127?term=radiosurgery&cond=Intracranial+Meningioma&draw=2&rank=1

## Background

Radiosurgery is a precise therapeutically effective radiation dose to an imaging-defined target [1]. Single session radiosurgery (sRS) represents the gold standard treatment for small and medium volume intracranial benign meningioma not suitable for surgical removal. The literature results ranged from 87% [2] to 100% [3, 4] in terms of both clinical stabilization and tumor growth control after 5 years from sRS. The treatment-related complications are also reassuring with low reported rates [5–7]. However, intracranial meningiomas are often close to critical structures or diagnosed when the volume is already large. In these cases, sRS could involve a high risk of treatment-related adverse effects, whereas multisession radiosurgery (mRS) can represent a viable treatment option as both exclusive and postoperative approach. By now, several retrospective studies focused on mRS for intracranial meningiomas have been published and all of them have reported promising results [8–13]. Although the effects are mostly encouraging, only long-term follow-up is able to investigate the impact of mRS in benign intracranial meningioma outcomes.

In this scenario, in 2011 we designed a prospective observational study to evaluate the safety and efficacy of radiosurgical multisession approach for treatment of both large and close to critical structures intracranial meningiomas. This phase II protocol can be appropriate to determine whether the multisession approach is worth to be studied through a phase III trials in the exclusive irradiation setting.

## Methods and design

### Study design

This is an observational phase II, prospective, monocenter, single-arm study focused on mRS for both large and/or close to critical structures intracranial meningiomas. Based on preliminary results, [8–12, 14] we hypothesized that a schedule of mRS delivering 25 Gy in 5 daily fractions could achieve a good local control and at the same time a low toxicity rate for large and/or critically located intracranial meningioma (lesions with a maximum diameter about or superior to 3 cm and/or located at around less than 3 mm from critical structures, e.g. near to chiasm, optic nerve, brainstem).

### Ethics

The Institutional Ethical Committee Board approved (2011 August 11th, number 415/2011) the study protocol, the declaration of Informed Consent and all case report forms (CRFs). The specific informed consent was provided and illustrated to each patient before the enrollment. This trial was registered to clinicaltrials.gov platform (Multisession Radiosurgery in Large Meningiomas –MuRaLM- identifier NCT02974127). We have been conducting it according to the principles of the Declaration of Helsinki and in accordance with the Medical Research Involving Human Subjects Act.

### Recruitment

Eligible patients who present at the Unit of Radiation Oncology of our Institute were recruited to the study. Eligibility requirements were:

### Inclusion criteria

Patients with radiological or histological diagnosis of large intracranial meningioma and/or critically located, then not suitable for sRS were eligible for our study. Other inclusion criteria were age ≥ 18 years old, KPS ≥ 70 and written informed consent.

### Exclusion criteria

Refusal of the patients to take part in the study; pregnant or lactating women; allergy to contrast medium; neurofibromatosis type II (NF2) diagnosis; atypical and malignant histologically confirmed meningiomas; diagnosis of optic sheath meningioma; and patients who had received prior radiotherapy at the same site.

### Dropout criteria

Criteria that will lead to the dropout of a patient before completion of this study may be any kind of treatment for medical reasons or impairment that will result in an interruption of radiotherapy. Furthermore, the patient’s desire to exit the study or withdrawal of consent as well as any unexpected serious adverse event (SAE) occurring during treatment or in follow-up that leads to early completion of the study will result in drop-out.

### Study aims and endpoints

The main aim is to evaluate the safety of the mRS in terms of prospectively scored adverse events (AEs), defined as stability or improvement of preexisting neurological deficits or as absence of new neurological deficits after irradiation. The secondary aim is to evaluate tumor response in terms of local control (LC), defined as stability or reduction of lesion volume.

### Assessment of the primary and secondary endpoints

#### Pretreatment evaluation

Pre-treatment evaluation comprises history taking interview, physical and neurological examination and radiological record evaluation. Karnofsky performance status (KPS) (Additional file 1: Appendix I), any prior brain surgery and/or brain radiation therapy have been registered. Moreover, when lesion involved optic pathways and steato-acoustic nerves, ophthalmological and/or audiometric evaluations were required.

As part of neurological examination, cranial nerve function has been recorded. For trigeminal nerve function assessment two parameters were used: the semi quantitative scales Barrow Neurological Institute pain intensity score (BPS) and Barrow Neurological Institute facial numbness score (BNS) [15]. The facial nerve function assessment was based on House-Brackmann (H-B) Facial Nerve Grading System for qualitative analysis [16]. For acoustic sensorineural assessment, two evaluations were registered: speech discrimination and audiometric threshold. For optic pathways assessment at least a bilateral visual field analysis and a visual acuity measurement were required. Any other deficits were recorded.

Table 1 summarizes prescribed pre-treatment investigations.

**Table 1:** Required pre-treatment investigations

Investigations	
Clinical history and physical exam	Clinical history, prior therapies, drug use, neurological examination
Hematology	Hemoglobin, white blood count, platelet count, creatinine
Adverse events	Baseline adverse event (baseline neurological symptoms and any post-surgery adverse events) recorded according to NCI CTCAE v. 4.0
KPS	See appendix 1
Visual acuity	
Visual field	
Speech discrimination	
Audiometric threshold	
BNS/BPS	
House Brackmann	
Radiology	Volumetric (1–3 mm thin slices) gadolinium-enhanced MRI

For unoperated patients the diagnosis of benign meningioma was clinical and radiological and was made by clinicians and expert neuroradiologists based on magnetic resonance imaging (MRI) and on additional computed tomography (CT) when required.

#### Follow-up evaluation

Patients have been being prospectively followed within the trial protocol after treatment for 5 years at least.

Post-treatment evaluations consist of neurological assessment as detailed before, audiometric evaluation and/or visual function assessment if required. Each AE has been being scored in accordance with the NCI Common Toxicity criteria, version 4.03 [17]. For local control follow-up, a gadolinium-enhanced thin slice (1 to 3 mm) brain MRI is required and T1-weighted volumetric post contrast sequence is mandatory as well as post contrast fat-saturation when anterior skull base lesion is under evaluation. To assess the tumor response, a volumetric analysis has been being performed by means of co-registration of each follow-up MRI on baseline MRI and contouring of the lesion on each post-treatment MRI, in order to ensure that the PTV and the post-irradiation lesions become spatially aligned and comparable.

Patients are scheduled for follow-up visits after 4 months from the treatment, every 6 months during the first 2nd and the 3rd years and thereafter every 12 months.

Tumor response has been defined and classified according to the following criteria:

- Complete response (CR) as a tumor disappearance.
- Partial response (PR) as ≥20% decrease in the tumor volumetric size.
- Stable disease (SD) as no change or any change different from the previous ones in tumor size.
- Progressive disease (PD) as increase in any tumor volumetric size outside the PTV or increase of tumor volume ≥ 20% confirmed at least on two consecutive MRI.

A checklist assessment has been constructed and reported in Table 2. The neurological assessment is detailed in Table 1.

**Table 2:** Intervention and assessment schedule for the MuRaLM trial

		Follow up			
Parameters	Recruitment	4 months	8 months	12 months	Every control
History, physical exam	X				
KPS	X	X	X	X	X
Neurological deficit assessment	X	X	X	X	X
AEs	X	X	X	X	X
Volumetric MRI/CT	X	X	X	X	X
Informed consent	X				

If death occurs or patients leave the study prior to 5 years, they are still included into the intention-to-treat population.

### Data collection

Based on General Data Protection Regulation (GDPR) guidelines, to ensure privacy respect during the clinical trial processes, an identification number has been provided for each patient. Clinical, neurological and radiological data has been being recorded on a case report form (CRF) and on an electronic database according to the good clinical practice guidelines (GCP) at baseline and in the follow-up period at each scheduled consultation [18].

### Sample size and statistical analysis

Assuming a one-sided score z test type I error of 5%, a power of 80%, an expected toxicity of the mRS at 5 years of 12% (H1, alternative hypothesis) vs a null hypothesis of 20% (H0), and assuming a loss to follow-up of no more than 20%, a sample of 170 patients is needed.

A description of patient characteristics at baseline will be provided in terms of percentages for dichotomous data, and means with standard deviations and medians with value ranges for continuous data. Primary analysis will be based on the score z test. Proper estimates with the corresponding 95% confidence intervals will be provided for primary and secondary outcomes at 5 years. Additionally, primary and secondary outcomes will be also analyzed as time to event variables using Kaplan-Meier method and Cox proportional hazards model (to adjust for potential confounders). STATA statistical software, version 15 (StataCorp. 2017. Stata Statistical Software: Release 15. College Station, TX: StataCorp LLC) will be used for the statistical analysis.

### Treatment planning and radiation technique

The mRS treatments were performed as previously described [19]. Briefly, the targets was delineated on high-resolution (1 mm thickness) contrast-enhanced computed tomography (CT) and on co-registered high-resolution MRI (1 mm thickness), using always T1 and T2-weighted contrast-enhanced sequences and when needed also using fat-saturated sequences. The tumour volume (TV) was identified based on these images as contrast-enhancing areas. We did not add any margins for clinical target volume (CTV) nor for planning target volume (PTV). Therefore, the PTV, CTV and TV (or GTV, gross tumour volume) were the same. All anatomical structures were contoured as organs at risk (OARs): brain, brainstem, retinas, eyes, lenses, optic nerves, optic pathways, cochleas, acoustic nerves, and skin. Organs at risk were contoured and adapted based on both CT scans and MRI.

The treatment plans were performed with an inverse planning method using non-isocentric technique (Multiplan Accuray Inc.). Based on literature data, [8–12, 14] we prescribed a dose of 25 Gy in 5 daily fractions to the isodose line encompassing 95% of the PTV (80% ± 5% prescription isodose line). We have considered as maximum dose to the OARs the prescription dose (25 Gy) at no more than 1 cc of the organ volume (D1cc ≤ 25 Gy). Unfortunately, no cognitive tests have been delivered. For this reason we did not analyzed the hippocampal dose for the analysis. The evaluation of setup and positioning verification was performed with an image-guided, real-time tracking system. According to our protocol, prophylactic dexamethasone (4 mg p.o. from the first day of treatment) was orally administered to all patients, unless they had some contraindications (e.g. insulin therapy). The patients maintained it and tapered it off during the 3 weeks following treatment. An H2-receptor antagonist was given in association with dexamethasone.

### Radiobiological perspective

Radiotherapy for intracranial meningiomas is an established treatment, as both fractionated stereotactic radiotherapy and radiosurgery approaches. The former usually delivers a dose of 54 Gy in 27–30 fractions while the latter delivers a dose of 13–14 Gy in single fraction. The two radiation settings are considered equivalent in terms of tumor control probability (TCP) [20]. Therefore, we can assume that benign meningiomas have an α/β ratio ranging from 1.75 to 3.8 Gy, [8, 9, 14, 21, 22] therefore the biologically equivalent dose (BED) of a 25 Gy in 5 fractions schedule is ranged between 61Gy and 87 Gy [9, 14]. In conclusion, due to the low α/β ratio, benign meningiomas benefits from hypofractionation and this schedule could represent the most appropriate choice [9, 14]. In this scenario, we decided to evaluate clinically this radiobiological hypothesis.

## Discussion

Meningiomas are the most common reported intracranial tumor (36.1%) and surgical resection is the gold standard for these lesions [22, 23]. However, a complete resection is not always achievable due to a high rate of possible complications (e.g. lesions near the critical structures) [24–28]. In these cases, RS can be a valid alternative to surgery [29]. While sRS can be an effective alternative to surgery for small meningiomas, [23] mRS could be a viable treatment in large or critically located meningiomas [14, 30, 31]. In fact, mRS seems to be comparable to sRS in terms of LC, in reducing potential complications, decreasing the occurrence of cognitive deficits [32] and the risk of symptomatic edema [33]. So far, several studies focused on a hypofractionated approach have been published with good results and low toxicity rate [8–14, 30, 31]. Nevertheless, safety and efficacy of this approach is not well characterized and the reported follow-up is too short for drawing any definitive conclusion [34].

Our results of previously published case series [14, 30, 31] seem to confirm these data, not only with a low risk of visual complications, but also improving visual function with good local tumor control in a limited but significant number of patients [23].

From this perspective, we carried out this observational prospective trial, to verify the safety and efficacy of a 25 Gy in 5 fractions mRS schedule for patients diagnosed with large or critically located meningioma in whom surgery and sRS were not indicated. According to the literature results, [14, 30, 31] we expect that AEs (dermatology/skin, neurology, and ocular/visual) are limited to grade 1 or 2, and that there are no grade 3 or higher events. The long-term follow-up will allow verifying the efficacy and safety of RS in multisession setting for treating critical intracranial meningioma. Due to the potential differences between large meningiomas and critically located ones, we will analyze efficacy and toxicity for both the entire cohort and separated subgroups. Moreover, the performed volumetric analysis will add some suggestions about the debated issue on how to conduct tumor response evaluation [23, 35].

## Supplementary information

**Additional file 1:** **Appendix I.** Karnofsky Performance Status Scale

## Data Availability

Not applicable.

## Abbreviations

BNS: Barrow Neurological Institute facial numbness score
BPS: Barrow Neurological Institute pain intensity score
CR: Complete response
CRF: Case report form
CT: Computed tomography
GCP: Good clinical practice
H-B: Facial nerve function will be graded on the House-Brackmann scale
KPS: Karnofsky performance status
MRI: Magnetic resonance imaging
mSR: Multisession radiosurgery
NCI: National Cancer Institute
NF2: Neurofibromatosis type II
OAR: Organ at risk
PD: Progressive disease
PR: Partial response
PTV: Planning target volume
RT: Radiotherapy
SD: Stable disease
sRS: Stereotactic radiosurgery
TPS: Treatment planning workstation
TV: Tumor volume
