# Supplementary figures and images for: Multisession radiosurgery for intracranial meningioma treatment: study protocol of a single arm, monocenter, prospective trial
Source: Radiat Oncol. 2020 Jan 30;15:26. doi: 10.1186/s13014-020-1478-7 (PMC6993396; doi:10.1186/s13014-020-1478-7)

**Appendix I: Karnofsky Performance Status Scale**


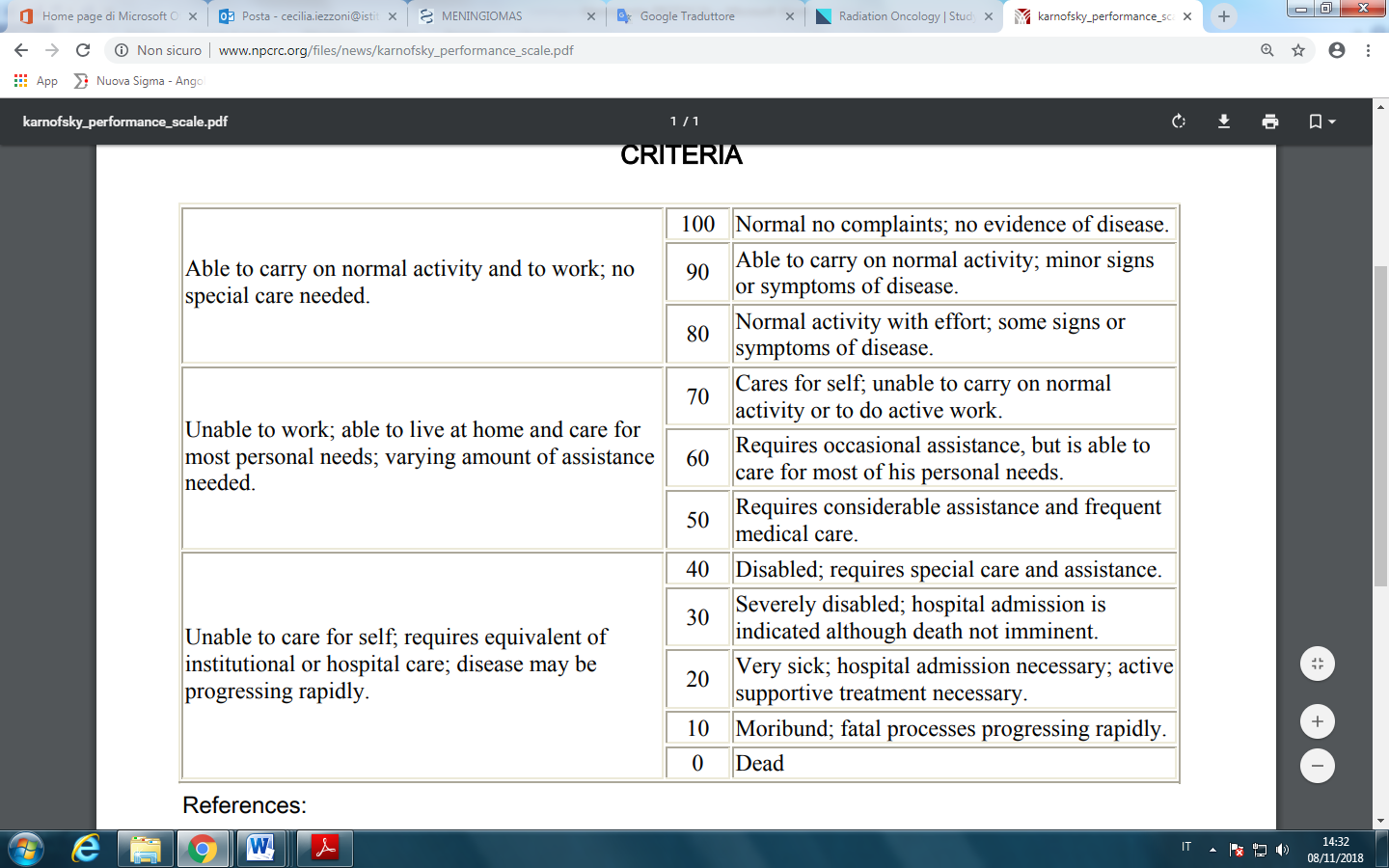

Supplement: Supplementary file 1 — Additional file 1: Appendix I. Karnofsky Performance Status Scale [file 13014_2020_1478_MOESM1_ESM.docx]
